# Long-Term Protection against HBV Chronic Carriage of Gambian Adolescents Vaccinated in Infancy and Immune Response in HBV Booster Trial in Adolescence

**DOI:** 10.1371/journal.pone.0000753

**Published:** 2007-08-15

**Authors:** Marianne A.B. van der Sande, Pauline A. Waight, Maimuna Mendy, Syed Zaman, Steve Kaye, Omar Sam, Abi Kahn, David Jeffries, Aveika A. Akum, Andrew J. Hall, Ebrima Bah, Samuel J. McConkey, Pierre Hainaut, Hilton C. Whittle

**Affiliations:** 1 Medical Research Council Laboratories, Fajara, The Gambia; 2 Gambia Hepatitis Intervention Study, International Agency for Research on Cancer, Lyon, France; 3 Department of State for Health, Banjul, The Gambia; 4 London School of Hygiene and Tropical Medicine, London, United Kingdom; Royal Free and University College Medical School, United Kingdom

## Abstract

**Background:**

Chronic infection with hepatitis B virus (HBV) arising in childhood is associated with hepatocellular carcinoma in adult life. Between 1986 and 1990, approximately 120,000 Gambian newborns were enrolled in a randomised controlled trial to assess the effectiveness of infant HBV vaccination on the prevention of hepatocellular carcinoma in adulthood. These children are now in adolescence and approaching adulthood, when the onset of sexual activity may challenge their hepatitis B immunity. Thus a booster dose in adolescence could be important to maintain long-term protection.

**Methods:**

Fifteen years after the start of the HBV infant vaccination study, 492 vaccinated and 424 unvaccinated children were identified to determine vaccine efficacy against infection and carriage in adolescence. At the same time, 297 of the 492 infant-vaccinated subjects were randomly offered a booster dose of HBV vaccine. Anti-HBs was measured before the booster, and two weeks and 1 year afterwards (ISRCTN71271385).

**Results:**

Vaccine efficacy 15 years after vaccination was 67.0% against infection as manifest by anti-HBc positivity (95% CI 58.2–74.6%), and 96.6% against HBsAg carriage (95% CI 91.5–100%). 31.2% of participants had detectable anti-HBs with a GMC of 32 IU/l. For 168 boosted participants GMC anti-HBs responses were 38 IU/l prior to vaccination, 524 IU/l two weeks after boosting, and 101 IU/l after 1 year.

**Conclusions:**

HBV vaccination in infants confers very good protection against carriage up to 15 years of age, although a large proportion of vaccinated subjects did not have detectable anti-HBs at this age. The response to boosting persisted for at least a year.

**Trial Registration:**

Controlled-Trials.com ISRCTN71271385

## Introduction

Recent estimates indicate that more than a third of the world's population, 2 billion people, have serological evidence of infection with hepatitis B virus (HBV). Around 360 million people are chronically infected; 60 million of whom reside in Africa. Up to a quarter of chronically infected individuals (depending on background mortality) may die as a result of the infection. It is estimated that at least half a million deaths per year are due to hepatitis B infection [1]. 10–15% of all adult male deaths in The Gambia are estimated to be related to hepatocellular carcinoma and chronic liver disease.

HBV persists in chronic carriers, who act as the main reservoir for infection. Transmission of the virus peaks in the first years of life, related to both perinatal transmission and horizontal transmission among young children, and again with the onset of sexual activity. To prevent infection with hepatitis B and its sequelae, routine infant vaccination against hepatitis B is recommended by the World Health Organization. Information on the longevity of protection resulting from the primary course in a highly endemic area, and the additional benefits (if any) of a booster would greatly facilitate decisions regarding the need to boost following infant vaccination.

The Gambia Hepatitis Intervention Study (GHIS) was launched in July 1986 with the objective of evaluating over a period of 40 years the effectiveness of hepatitis B vaccination in the prevention of hepatitis B infection, chronic liver disease and primary liver cancer in a population at high risk. Hepatitis B vaccination was introduced into the national immunisation programme using a ‘stepped wedge’ trial design. Every few months, one of the existing vaccinations centres selected at random would start administering hepatitis B vaccination in addition to the other infant vaccinations, until countrywide coverage was achieved [2]. Between 1986 and 1990, about 60,000 children received plasma-derived hepatitis B vaccine (HBVvax®; Merck, Sharpe & Dohme) with the other EPI vaccines; and about 60,000 children received only the regular vaccines in the first year of life. Subsequent longitudinal and cross sectional surveys of selected samples of the two groups were carried out over the first 10 years of life.

Follow-up showed that the immune response to vaccination in infancy was excellent, despite antibody levels waning over the years. At 9 years of age, 31% of children had anti-HBs titres below 10 IU/l, which is the minimum concentration needed to ensure protection. Another 40% had levels of antibody ranging from 10–100 IU/l. However, protection against hepatitis B infection and against chronic carriage in the vaccinated group remained relatively constant between the age of 4 years and of 9 years (being 83% and 94% respectively), in spite of this waning of antibody [3]. Transient infections, which may boost both cellular and humoral immunity, were noted in one third of infected children. This continued exposure to the virus among vaccinees has also been noted in other populations [4]. A small long-term study from Senegal with a large loss to follow-up reported efficacies nine to twelve years after vaccination of 63% against infection and 87% against chronic carriage [5]. One small study in China also assessed vaccine efficacy after 15 years of age [6]. Since exposure to infection is limited at this age, it is unclear whether or not this low level of antibody confers an increased risk of infection and in particular in the risk of becoming a chronic carrier following a subsequent challenge, eg through sexual exposure. Immunological memory persists in spite of non-detectable antibodies and can produce an adequate response within days. However, it is not known how long such immunological memory persists, and the delay prior to activation of the immune system may offer a window of opportunity for transmission of the virus [7].

The rise in anti-HBs antibodies following boosting can be quantified. The subsequent decay can be modelled to estimate the number of years gained with protective serum antibody levels >10 IU/l, and thus to estimate the number of anti-HBc antibody conversions averted.

In a recently completed study in two small Gambian villages, the protection provided by vaccination in early life against infection remained stable between the ages of 1–4 (93%) to 10–14 years (92%), but started to decline at older ages (81% among the 15–19 years old, 71% among the 20–24 years olds). Protection against carriage showed a similar trend (still very high at 98% at 10–14 years, 96% at 15–19 year, and 91% among the 20–24 year olds) [8]. This study used historical control data to estimate vaccine efficacy. The decline in vaccine efficacy during adolescence may coincide with the onset of sexual activity. We had already shown that time since vaccination and undetectable antibody levels following vaccination (<10 IU/l) were the most powerful risk factors for breakthrough infection (p<0.0001 in both cases) in adolescence [9]. Previously we had shown that undetectable antibody levels in the year preceding sero-conversion to anti-HBc were also associated with a much increased risk for infection in children at 7 years of age [10].

This study aimed to evaluate long-term vaccine efficacy of infant vaccination in a large adolescent sample, as a follow-up within the original GHIS trial, representative of the general population, using concurrent unvaccinated controls, and to assess the immune response to an adolescent booster dose after 2 weeks and one year in the same population.

## Methods

### Participants

Vaccine efficacy and response to boosting were assessed in a cross-sectional sample of individuals originally recruited into the GHIS as infants. Recruitment was limited to the catchment areas of the five health centres in the study where vaccination started only in 1989. Participants were eligible if born between 1 July 1988 and 31 December 1989, and if they could be identified and matched with the original GHIS database.

In each catchment area, a random sequence of enumeration areas was generated. Within each enumeration area, fieldworkers went from compound to compound recording identification details of potential recruits until the target number for a catchment area was reached.

**Figure 1:**
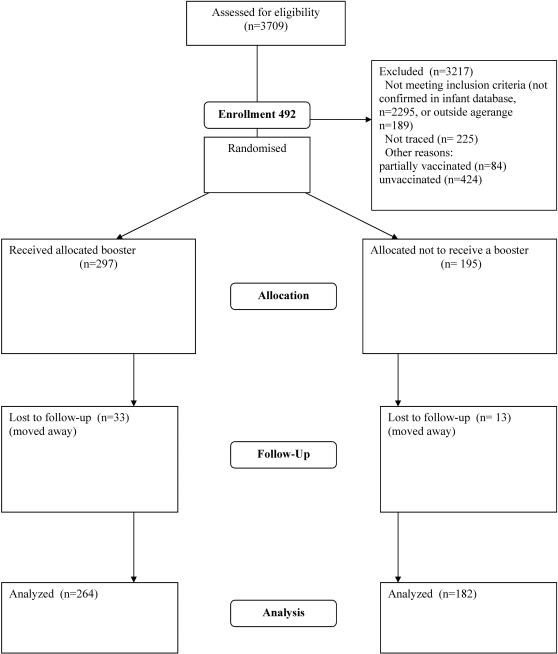
Flowchart HBV booster allocation.

Fieldworkers and nurses revisited participants who had been matched with the original GHIS database to ask for a venous bloodsample. A random selection of 60% of fully vaccinated participants was assigned to receive a booster dose of hepatitis B vaccine (Euvax®), followed by an additional blood sample to measure anti-HBs antibodies level 2 weeks afterwards. The protocol for this trial and supporting CONSORT checklist are available as supporting information; see CONSORT S1 and Protocol S1. All vaccinated subjects, whether boosted or not, were asked for a final sample to determine anti-HBs antibodies one year after the first blood sample (figure 1).

### Objectives and Outcomes

The primary objective of the follow up was to establish vaccine effectiveness against anti-HBc and HBsAg positivity. The primary objective of the nested RCT was to establish the short term (2 weeks) and medium term (1 year) anti-HBs response to a HBV boost in adolescence. Anti-HBs antibody levels two and 52 weeks after a booster dose enable an assessment of the duration of a response, as well as assessing the proportion of vaccines with and without an anamnestic response.

### Laboratory

Anti-HBc and anti-HBs antibodies were detected using a commercial EIA (ETI-AB-Corek Plus and ETI-AB-AUK respectively, DiaSorin, Saluggia, Italy) according to the manufacturer's instructions. The level of detection of anti-HBs was 10 IU/l.

Samples which were anti-HBc positive, were tested for HBsAg by Determine^TM^ HBsAg (Abbott Laboratories), a visually read, qualitative immunochromatographic assay. All those who tested HBsAg positives were retested after 6 months to confirm carrier status. Samples which were HBsAg positive, were tested for HBeAg using an enzyme immunoassay [EIA] (Equipar Diagnostici, Saronno (Va), Italy).

### Definitions

If one or two doses of HBV were recorded in the GHIS database the participant was considered partially vaccinated; if three or four doses were received this was defined as fully vaccinated.

Infection was defined by the presence of anti-HBc with at least 30% inhibition, as stipulated by the manufacturer. If infection was found in a person vaccinated in infancy, this was considered a breakthrough infection. Chronic carriage was defined as testing HBsAg positive at both the initial and the 12 month follow-up survey.

An early anamnestic response to the booster dose was defined as a four fold or greater rise in anti-HBs antibody within two weeks of the booster dose, or for children with no detectable antibody in the first sample, an anti-HBs antibody level ≥10 IU/l within two weeks of the booster dose, in line with recently published CDC methods [11]. A long-term anamnestic response was defined as at least a two fold rise in anti-HBs antibody one year after boosting, or if no detectable antibody response at baseline, an anti-HBs antibody level of ≥10 IU/l.

### Sample size

Based on the vaccine efficacy data from previous follow-up of the GHIS after 9 years (83% against infection, 94% against carriage) [3] and assuming that among the unvaccinated group the prevalence of infection had risen to 75% and the prevalence of carriage to 12%, and that in the vaccinated group anti-HBc prevalence had risen to 20% and persistent HBsAg positivity to 2%, we calculated that we needed to recruit 500 participants in each group (vaccinated and unvaccinated) to estimate a vaccine efficacy against infection of 73% (CI 68–78) and against carriage of 83% (CI 70–93). Increasing the sample size had only a minor effect on the width of the confidence intervals. Different assumptions result in different efficacy estimates, but do not significantly affect the width of the confidence intervals.

As we assumed that loss to follow-up after one year might be larger in the vaccinated group randomised to a booster (due to the 2 week bleed among the boosted group), we calculated that with a distribution of 300 boosted and 200 not boosted, and assuming a 20% loss to follow-up, we would be able to measure antibody responses after 52 weeks in 200 boosted and 160 unboosted vaccines, which would give us over 90% power to detect a significant difference in a protective level of antibodies (defined as anti-HBs>10 IU/ml) between the boosted and unboosted group.

This sample size was adjusted based on assumptions of percentage which could be matched to the original database (50%), matched participants found on follow up (90%) and then consenting to be bled (90%; 80% for the bleed two weeks after the booster; 60% for the bleed one year later). Thus, a total of 3500 potentially eligible 15 year olds were estimated as necessary for initial recruitment. For each of the five catchment areas, a sample size proportional to size was calculated.

### Matching and data management

A questionnaire collected personal and demographic information from all potential recruits. Items included names for both participants and parents, date and place of birth, Infant Welfare Card (IWC) number, ethnic group and date of previous hepatitis vaccination if recorded on the IWC.

Data were double entered and validated directly into a table in an Access database (Microsoft) also containing a data table with details of the original GHIS cohort. A sequence of queries was run to match recruits with GHIS participants. Data for non-matching recruits were imported to Reclinc2 (IARC) where further non-specific matching techniques were used involving soundex and cross matching of all family names.

Matched recruits were defined as confirmed or probable matches. Confirmed matches by definition had the same combinations of IWC number and date of birth whilst names were generally similar with allowances for differences in spelling or abbreviations. For the purposes of the follow up only confirmed matches were included.

Laboratory data were exported directly from the assay reader to Excel (MICROSOFT) and directly imported into the study database.

### Randomisation

In each area from which participants were recruited, stratified by sex, a random 60% sample was identified, using computer generated lists. Laboratory staff responsible for the outcome measurements were blinded to the allocation.

### Statistical methods

Data were analysed using Stata 8.0 (Stata Corp, Texas, USA). Surface antibody levels were log-transformed and a Geometric Mean Concentration (GMC) calculated where a response was measured (anti-HBs ≥10 IU/l). Vaccine efficacy was calculated as 1-(prevalence in the vaccinated/prevalence in the unvaccinated). Statistical significance of differences between groups was assessed with a Pearson Chi-square test or by Fisher's exact test, as appropriate. A p-value<0.05 was considered to denote statistical significance.

Logistic regression was used to estimate crude and adjusted odds ratios for an anamnestic response after boosting by number of doses received in infancy and by core-positivity prior to the boost.

### Ethics

The study was approved by the Gambia Government/MRC Ethics Committee. All participants and their guardians gave signed informed consent. The incorporated trial assessing the effect of boosting or not among infant vaccinnees (ISRCTN71271385) was monitored to ensure it complied with Good Clinical Practice guidelines. Written feedback of results was provided to all participants.

## Results

### Participants

Between February and July 2004, 3709 potentially eligible participants were identified in the selected areas. For 2147 (57.9%) a satisfactory match was found in the original database. An absolute match was confirmed for 1414, of which 1225 were of the correct age. Of these, 1000 (81.6%) were traced and consented to a blood sample being taken. Of these, 492 (49.2%) had been fully vaccinated (320, 65.0% of them with 4 doses, the remainder with 3 doses), 84 (8.4%) partially, and 424 (42.4%) were not vaccinated as infants.

Of the fully vaccinated participants who consented to a blood sample being taken, 297 out of 492 were randomly allocated to receive a booster dose. Of these 255 out of 297 (85.6%) donated a sample after two weeks. After one year, a sample was received from 264 out of the 297 (88.9%) of those boosted and 182 out of 195 (93.3%) of the unboosted subjects (figure 1).

Adolescents not vaccinated in infancy were on average slightly older than the vaccinated infants. There was a balanced distribution within the boosted and unboosted participants (table 1).

**Table 1:** Baseline characteristics of participants identified.

	Vaccinated			Partially vaccinated	Unvaccinated
	All	Boosted	Not boosted		
Recruited	492	297	195	84	424
Female n (%)	272 (55.3%)	162 (54.6%)	110 (56.7%)	53 (63.1%)	228 (53.8%)
Mean age (sd)	14.9 (0.4)	14.9 (0.4)	14.9 (0.4)	15.0 (0.4)	15.6 (0.3)
Ever jaundiced	39 (7.9%)	24 (8.1%)	15 (7.7%)	4 (4.8%)	38 (9.0%)

### Vaccine efficacy

Of the unvaccinated population, 226 out of 424 (53.3%, 95% CI 48.4–58.1) were anti-HBc positive; 19 out of 84 (22.6%, 95% CI 14.2–33.0) of the partially vaccinated group, and 87 out of 492 (17.7%, 95% CI 14.4–21.3) of the fully vaccinated children. Among the fully vaccinated children, there was no difference in prevalence of anti-HBc positivity among those who had received three or four doses in infancy (16.8% vs 18.1%, p = 0.7); nor among the partially vaccinated children who had received one or two doses (25.0% vs 21.2%, p = 0.7). Thus, 15 years after vaccination, vaccine efficacy against infection of the fully vaccinated children versus unvaccinated children was 67.0% (95% CI 58.2–74.6). Vaccine efficacy against infection for the partially vaccinated children versus unvaccinated children was lower at 57.7% (37.2–76.8) (table 2).

**Table 2:** Vaccine efficacy against infection (anti-HBc positive) and chronic carriage (HBsAg positive) among 15 year old Gambians, according to hepatitis B infant vaccination status.

	Fully vaccinated	Partially vaccinated	Unvaccinated
Infection (n/N, % 95% CI)	87/492 (17.7%, 14.4–21.3)	19/84 (22.6%, 14.2–33.0)	226/424 (53.3%, 48.4–58.1)
VE (95% CI)	67.0 (58.2–74.6)	57.7 (37.2–76.8)	Ref
Chronic carriage (n/N, % 95% CI)	2/492 (0.4%, 0.04–1.5)	1/84 (1.2%, 0.03–6.5)	51/420 * (13.2%, 10.1–16.8)
VE (95% CI)	96.6 (91.5–100)	90.1 (69.9–100)	ref

* four participants were HBsAg positive during the first screening, but could not be traced to confirm chronic carriage

VE: vaccine efficacy

CI: confidence interval

Of those infected, 56 of the unvaccinated, one of the partially vaccinated and two of the fully vaccinated children were HBsAg positive. To assess if these were chronic carriers, a follow-up sample was collected one year later. Four out of 56 unvaccinated HBsAg positive participants could not be traced after one year to determine whether they were chronic carriers. All but one of the remaining 52 unvaccinated subjects who could be traced were confirmed as chronic carriers. Excluding the 4 unvaccinated subjects lacking confirmation of chronic HBsAg positivity, vaccine efficacy of the fully vaccinated against carriage was 96.6% (96% CI 91.5–100), and for the partially vaccinated versus unvaccinated was 90.1% (95% CI 69.9–100) (table 2).

### Baseline anti-HBs data and response to a booster dose

Baseline anti-HBs level could be determined for 570 subjects. At the onset of the study, 392 out of 570 (68.8%, 95% CI 64.8–72.6) participants had undetectable surface antibody levels, significantly more among unvaccinated subjects (99/126, 78.6%, 95% CI 70.4–85.4) than among infant-vaccinated subjects (254/382, 66.5%, 95% CI 61.5–71.2, p-value 0.01), but did not differ between the vaccinated groups randomised to a booster dose or not (table 3). The percentage of individuals with detectable antibody was highest among the partially-vaccinated group (23/62; 37.1% 95% CI 25.1–100). The overall GMC was 31.6 IU/l (95% CI 26.7–37.4), and did not differ significantly between the groups (table 4).

**Table 3:** Number (%, 95% CI) of vaccinated and boosted subjects in each anti-HBs category prior to boosting, and 2 weeks and 1 year afterwards.

	Pre boost			2/52 Post boost	12/12 post boost	
Anti-HBs in IU/l	Unvaccinated	Vaccinated to be boosted	Vaccinated not to be boosted		Boosted	Not-boosted
n	126	225	157	217	264	182
<10	99 (78.6%) (70.4–85.4)	148 (65.8%) (59.2–72.0)	106 (67.5%) (59.6–74.8)	7 (3.2%) (1.3–6.5)	57 (21.6%) (16.8–27.0)	139 (76.4%) (69.5–82.3)
10–99	25 (19.8) (13.3–27.9)	65 (28.9%) (23.1–35.3)	46 (29.3%) (22.3–37.1)	24 (11.1%) (7.2–16.0)	117 (44.3%) (38.2–50.5)	34 (18.7%) (13.3–25.1)
100–999	2 (1.6) (0.2–5.6)	11 (4.9%) (2.5–8.6)	5 (3.2%) (0.1–7.3)	107 (49.3%) (42.5–56.2)	77 (29.2%) (23.8–35.1)	9 (5.0%) (2.3–9.2)
≥1000	0	1 (0.4%) (0.01–2.5)	0	79 (36.4%) (30.0–43.2)	13 (4.9%) (2.6–8.3)	0

**Table 4:** Proportion (95% confidence interval) with detectable surface antibody levels and GMC of participants by infant vaccination and adolescent boosting status.

	Vaccinated				Partially vaccinated	Not vaccinated
	All	Boosted	Not boosted	p-value		
**Baseline**	382/492 (77.6%)	225/297 (75.7%)	157/195 (80.1%)		62/84 (73.8%)	126/424 (29.7%)
Anti-HBs ≥10 IU/l	128 (33.5%) (28.8–38.5)	77 (34.2%) (28.0–40.8)	51 (32.5%) (25.2–40.4)	0.7	23 (37.1%) (25.1–50.0)	27 (21.4%) (14.6–29.6)
GMC if anti-HBs detected	31.4 (26.0–38.0)	34.8 (26.9–44.9)	27.0 (20.2–36.0)	0.2	45.8 (22.6–92.6)	23.6 (17.4–32.0)
**2 weeks** after boost	-	217/297 (73.1%)	-		-	-
Anti-HBs ≥10 IU/l	-	210 (96.8%) (93.5–98.7)	-		-	-
GMC if anti-HBs detected	-	498.3 (424.0–585.6)	-		-	-
**1 year** after boost	446/492 (90.6%)	264/297 (88.9%)	182/195 (93.3%)		-	-
Anti-HBs ≥10 IU/l	250 (56.1%) (51.3–60.7)	207 (78.4%) (73.0–83.2)	43 (23.6%) (17.7–30.5)	<0.001	-	-
GMC if anti-HBs detected	76.2 (64.0–90.7)	89.5 (74.0–108.3)	35.1 (24.2–51.0)	0.0001		

Two weeks after the booster dose, 7/217 (3.2%, 95% CI 1.3–6.5) of all boosted participants had undetectable anti-HBs, versus 148/225 (65.8%, 95% CI 59.2–72.0) prior to the boost; while 79/217 (36.4%, 95% CI 30.0–43.2) had an anti-HBs level above 1000 IU/l (table 3). Of the participants with undetectable antibodies at baseline, 7/114 (6.1%, 95% CI 2.5–12.2) still had no detectable anti-HBs response 2 weeks after the boost.

For 181 out of 297 of the boosted participants anti-HBs responses were measured at baseline and two weeks after the booster. Of these, 167 (92.3%, 95% CI 87.4–95.7) showed an early anamnestic response. In this group the GMC had increased from 36 IU/l (95% CI 27–47) to 524 IU/l (95% CI 441–621). Among the vaccinated boosted subjects who were anti-HBc positive at baseline, 12/17 (70.6%, 95% CI 44.0–89.7) showed an early anamnestic response, compared to 155/164 (94.5%, 95% CI 89.8–97.5) of boosted subjects who were anti-HBc negative at baseline(p<0.001).

One year after the boost, 57 out of 264 (21.6%, 95% CI 16.8–27.0) of boosted individuals had undetectable surface antibodies (table 3). Of the 7 participants who did not have a response two weeks after the booster dose, five still had undetectable antibody levels, but two now had a measurable anti-HBs concentration (14.1 and 101.7 IU/l respectively).

A long-term anamnestic response was detected in 137 out of 197 (69.5%, 95% CI 62.6–75.9) of boosted participants. Among the 16 vaccinated boosted subjects who were anti-HBc positive, 6 (37.5%, 95% CI 15.2–64.6) showed a long-term anamnestic response, compared to 131 out of the 181 (72.4%, 95% CI 65.2–78.7) of those who had remained anti-HBc negative (p = 0.001).

In total, anti-HBs responses were evaluated at all three points (at baseline before boosting, two weeks and one year afterwards) in 168 of the boosted subjects. The GMC of this group at baseline was 38 IU/l (95% CI 29–50), which increased after two weeks to 524 IU/l (95% CI 438–626) and fell to 101 IU/l (95% CI 79–129) at 1 year. Among the 144 unboosted participants bled at baseline and one year afterwards, the GMC remained similar at 29 and 28 IU/l respectively.

Anti-HBc positivity was associated with significantly reduced odds ratio (OR) of having an early and a long-term anamnestic response. In a multivariable analysis, adjusting for sex, area, and number of doses, the OR was further reduced. Anti-HBc-positivity was also associated with a significantly reduced OR of having an anamnestic response after one year. Those who had received four doses in infancy had an increased OR for an anamnestic response after two weeks which remained borderline significant after one year compared to those who had received three doses (table 5).

**Table 5:** Crude and adjusted odds ratios (OR, 95% CI) for a short term (2 weeks) and a long term (1 year) anamnestic response after boosting.

	Short term		Long term	
Variable	Crude	Adjusted	Crude	Adjusted
Anti-HBc positive vs anti-HBc negative	**0.1** (0.04–0.45)	**0.05** * (0.01–0.3)	**0.2** (0.08–0.7)	**0.2** * (0.07–0.6)
Four doses of vaccine vs three doses of vaccine	2.5 (0.8–7.6)	**5.2** ** (1.3–20.7)	1.6 (0.9–3.0)	**1.9** ** (1.0–3.8)

* Odds ratio's adjusted for sex, area, number of doses

** Odds ratio's adjusted for sex, area, core-positivity

### Adverse events

No adverse events related to receiving a booster dose were noted.

## Discussion

Within the GHIS population, vaccine efficacy of infant HBV vaccination against carriage has remained high at 15 years of age, as was also observed in a previous study at 9 years of age [3] (94% and 97% respectively), while vaccine efficacy against infection declined from 82% at 9 years of age to 67% at 15 years of age. In this study, over two thirds of the 15 year olds did not have detectable anti-HBs levels, which is similar to the previous observations at 9 years of age. As expected, following a booster vaccination, a marked increase in surface antibody was seen; with 97% of boosted participants having detectable antibody after 2 weeks, and nearly 80% still a year after the booster, compared to a third prior to the boost. This low pre-boost antibody level suggests that either limited natural exposure to HBV with subsequent subclinical boosting had occurred during the early teenage years; or that natural boosting did not result in a sustained anti-HBs response.

In spite of the excellent response to boosting in general, 3% of boosted participants did not mount a detectable antibody response following a booster dose of HBV. It is possible that these individuals may have been primary non-responders as a study in two rural Gambian villages showed that two months after the last infant vaccination, 5% of children did not mount a detectable anti-HBs response and were therefore considered primary non-responders to the HBV vaccine [12]. On the other hand, of the 7 non-responders among the boosted subjects, two participants did show an effective response a year later. These 2 may have mounted a primary response to this single dose of vaccine. It is also possible that sub-clinical exposure had occurred during the year, which boosted the antibody response. Alternatively, some presumed non-responders could actually be slow responders, corresponding with a low rate of memory accumulation [7].

It is remarkable that vaccine efficacy against chronic carriage among the group who were only partially vaccinated was rather similar to that of the group who had been fully vaccinated. Several studies have already shown that adolescents and adults vaccinated with two or even with one dose, had similar short-term antibody responses to those vaccinated with 3 doses [13], [14], [15], [16], [17], [18], [19]. We have also recently demonstrated that children vaccinated with two doses rather than three, had similar vaccine efficacy against infection and carriage after 4 to 7 years (submitted). This study now suggests that those only partially vaccinated against HBV in infancy not only have similar antibody responses as those who received at least 3 doses, but may have also a similar long-term vaccine efficacy as those fully vaccinated for up to 15 years. Nevertheless, it should be noted that the lower limit of the confidence interval is only around 70% versus the greater than 90% in the fully vaccinated; this study is too small to establish equivalence of protection.

These findings are of relevance for the understanding of the long term protection of HBV vaccine in infancy, in a population still heavily exposed to HBV as suggested by similar anti-HBs antibody levels at baseline in both vaccinated and unvaccinated groups, and by the observation that over half of the unvaccinated subjects were positive to anti-HBc.

This study also showed that it is possible to trace records for a large proportion of infants 15 years after recruitment, in a sub-Saharan setting in the absence of consistent identifying documentation. The main outcome of the GHIS study is only expected to occur 40 years following the start of the infant vaccination programme, which is dependent on the ability to trace patients with hepatocellular carcinoma in the GHIS database to ascertain their vaccination status (GHIS). Using advanced iterative search strategies, nearly 60% of adolescences who reported that they were in the correct age range of the GHIS were identified in the database, although for a third of this group, it was not possible to confirm the match mainly due to the recruit not knowing their date of birth. It is expected that most of this group could be fully matched based on the foot- and palm prints, which were collected from the infants at the start of the trial to enable final matching.

In view of the low levels of circulating antibody in adolescence, there is uncertainty if the high vaccine efficacy against carriage can be maintained once infant vaccines become exposed to HBV via sexual routes. In fact, despite high natural exposure to circulating HBV infection, it is not clear whether natural boosting can sustain protection or not over time. The excellent short-term response to boosting suggests immunological memory is well maintained [20]. A recent consensus statement based on published evidence concluded that no booster was required for 15 years after primary vaccination [21]. However, this was based on a single publication from China where 54 unvaccinated and 52 vaccinated children were followed up at this age [6]. The authors themselves acknowledged that their study had serious limitations especially due to the incomplete follow-up and the alteration of the original cohort. Furthermore, since risk of infection associated with adolescence may differ in other areas of the world, it is unclear how far this finding can be generalised. Therefore, it remains to be evaluated whether a memory response would be sufficient to prevent infection and carriage following sexual exposure in adulthood. Our study showed that nearly 20% of those fully vaccinated had become anti-HBc positive, which gave a significant reduction in the likelihood of having a short term and a long-term anamnestic response. Once more vaccines against sexually transmitted infections become available (such as HPV and HSV) adolescents are likely to be a major target group for them. HBV booster vaccination will be a major candidate for evaluation.

## Supporting Information

CONSORT S1 Consort Checklist
(0.05 MB DOC)

Protocol S1 Trial Protocol
(0.09 MB DOC)
