# Supplementary material for: Long-Term Protection against HBV Chronic Carriage of Gambian Adolescents Vaccinated in Infancy and Immune Response in HBV Booster Trial in Adolescence
Source: PLoS One. 2007 Aug 15;2(8):e753. doi: 10.1371/journal.pone.0000753 (PMC1940311; doi:10.1371/journal.pone.0000753)
Supplement: Protocol S1 — Trial Protocol (0.09 MB DOC) [file pone.0000753.s002.doc]

To:	Dr Robin Bailey

Chairman SCC

##	MRC Laboratories

Fajara

Fajara, 17 November 2003

Dear Dr Bailey,

Re: modification HBV VE study, SCC 886/843 – L2003.64

In July 2002, the SCC approved our proposal for a randomised controlled trial (RCT) on the efficacy of a hepatitis B booster dose in adolescence for subjects vaccinated as part of the GHIS (GHIS 1987), using core conversion and carriage as endpoints (SCC886/843). This proposal was submitted to MRC for funding, but although an alpha-A rating was awarded, funding could not be released. Pending identification of alternative funding, we proposed to already proceed with the Vaccine Efficacy (VE) component from this RCT, in order not to loose the window of opportunity to assess VE in adolescence at the assumed age of sexual onset (L2003.64). This modification was approved by the SCC and is still awaiting funding, which we hope will be released soon, as the youngest GHIS participants are now 14 years of age.

In view of our unsuccessful attempts so far to secure the budget for the above RCT, and the aging of the GHIS cohort, we now propose to modify the approved VE study, by offering a HBV boost to a subgroup of vaccines in order not to loose the unique opportunities of the GHIS cohort to understand the impact of boosting in adolescence. Adding a boost will enable us to assess the magnitude and duration of immune responses (measured two and 52 weeks after the boost) and estimate the proportion of infant vaccinees that do not have evidence of immune memory in adolescence. This study will complement the study submitted to this SCC by Dr McConkey *et al*, aimed at a detailed exploration of cellular and humoral immune responses following boosting, as well as a detailed study of primary and secondary immune responses to HBV vaccine.

Briefly, the immune response to HBV vaccination can be assessed by determining the B cell responses (quantified in level of anti-HBs in serum). A serum antibody level above 10IU/l is defined as a protective response. The antibody level is highest in the months following vaccination, and decreases exponentially with time. The rise in anti-HBs following boosting can be quantified, and the subsequent decay can be modelled to estimate the number of years gained with protective serum antibody levels >10IU/l, and thus to estimate the number of core antibody conversions averted.

This loss of an immune response with time since vaccination is confirmed by findings in the follow up of the GHIS and the Keneba-Manduar cohort, where protection against infection decreased with age. Preliminary data from the 2003 follow-up of the Keneba Manduar cohort show that the percentage of participants who are core antibody positive increased with age: from 4.1% in the 1-4 year olds, to 24.2% in the 20-24 year olds. Time since vaccination and undetectable antibody levels following vaccination (<10 IU/l) were the most powerful risk factors for core conversion in the 2003 survey, as observed before (Whittle *et al 2002*). Previously we had shown that undetectable antibody levels in the year preceding conversion were also associated with a much increased risk for infection in children at 7 years of age (Jack *et al* 1999). It is essential to confirm these findings on a larger and more representative sample from the population. GHIS recruits live in an ‘open’ vaccine environment where vaccine coverage might be quite different than that surrounding the Keneba Manduar cohort.

Measuring anti-HBs two and 52 weeks after a boost will enable us to assess the duration of a response, as well as assessing the proportion of vaccines with and without an anamnestic response. An anamnestic response will be defined as an at least four fold rise in anti-HBs within two weeks of the booster dose, or for children with no detectable antibody, an anti-HBs above 10IU/l within two weeks of the booster dose, in line with recently published CDC methods (Williams *et al* 2003).

Methods

Methods will be similar to those in the original proposals. However, in view of the preliminary 2003 VE data from Keneba-Manduar follow up, and as previous VE studies of the GHIS cohort did not show a difference in VE in urban and rural area (Viviani *et al* 1999) we now propose to defer a urban-rural stratification. This will enable us to reduce the sample size to 500 vaccinees and 500 unvaccinated controls for the VE assessment. In view of the passing of time and the need to identify unvaccinated participants in the same age-range, eligible participants for the VE study will be recruited from the catchment areas of the centres in the study area where GHIS HBV vaccination was introduced in 1989 only (Banjul, Serekunda, Brufut, Banjulunding, Sukuta). For all 13-15 year olds identified in the GHIS database, a 5 ml sample of venous blood will be collected in an EDTA tube. Immediately after this sample has been collected, 300 consenting vaccinees will be randomised to receive an adult dose of the recombinant HBV vaccine currently in use in the EPI program. Plasma will be separated and stored within 24 hours of collection. Anti-HBc and anti-HBs will be measured by EIA; samples testing positive for anti-HBc will also be tested for HBsAg (Sorin Diagnostics). All boosted vaccines will be asked for another 5 ml venous sample to be collected in EDTA two weeks after boosting and all vaccinnees will be asked for a 5 ml sample 52 weeks after the baseline sample was collected to assess persistence of anti-HBs. Unboosted vaccinnees will be offered a boost after the 52 week sample. To account for the effect of natural boosting, intra-group differences will be compared between boosted and unboosted infant vaccinnees. Subjects with a HbsAg positive test will be traced and asked for another sample after 6 months, to determine whether or not they are chronic carriers, as described. Unvaccinated participants who are still susceptible to infection, will be offered a full course of HBV vaccination. Study procedures will be monitored, and an independent external advisor will be identified.

Sample size.

A. *VE*. As explained in L2003.64, in order to obtain estimates of VE with reasonably narrow confidence intervals, assuming that among the unvaccinated group the prevalence of infection has risen to 75% and the prevalence of carriage to 12%, and that in the vaccinated group anti-HBc prevalence has risen to 20% and persistent HBsAg positivity to 2%, we need to recruit 500 participants in each group, to estimate a vaccine efficacy against infection of 73% (CI 68-78) and against carriage of 83% (CI 70-93). Increasing the sample size has only a minor effect on the width of the confidence intervals. Different assumptions result in different efficacy estimates, but do not significantly affect the width of the confidence intervals.

B. *Boosting*. This is primarily an explorative study, aimed at determining the magnitude and persistence over time of an increase in surface antibody, and at estimating the proportion of infant vaccinees who do not have evidence of immune memory in adolescence as shown by failure to mount an anamnestic secondary response to boosting. We assume that of the 300 vaccinees randomised to boosting, 250 will be vaccinated and have a follow-up bleed after 2 weeks. If we find that 10% of 250 infant vaccinated infants have lost memory and react with a primary response, 95%CI on this estimate are 7-14%. If this is the case for 25%, 95%CI on the estimate will be 20-31%. It is critical that we have a reasonably narrow estimate of the proportion of adolescents who have lost memory, in particular as we will not be able to exclude primary non-responders (estimated at 1-2%).

Assuming 20% loss to follow-up over a 1-year period, we would be able to measure antibody responses after 52 weeks in 200 boosted and 160 unboosted vaccines, which would give us over 90% power to detect a significant difference in a protective level of antibodies (defined as anti-HBs>10IU/ml) between the boosted and unboosted group, if we assume that 5% of boosted and 40% of the unboosted group have anti-HBs below 10IU/ml at 52 weeks. By analysing the distribution of the antibody data (e.g. by using a kernel density estimation) of boosted and unboosted subjects at 52 weeks we will obtain the necessary information for a precise sample size calculation for further comparative studies.

In summary, we aim to assess both VE and the impact of boosting in adolescence, i.e. the magnitude and duration of the boost in immune responses, in this unique cohort just prior to the assumed age of sexual onset and increased risk of HBV infection. As stated before, a major additional benefit of this evaluation will be further testing and development of the GHIS cohort and database, which is an essential component for the long-term GHIS objective relating to the efficacy of infant HBV vaccination in preventing hepatocellular carcinoma. Furthermore, this study will offer an opportunity to increase the number of samples available for Branwen Hennig’s externally funded ongoing study in the role of host genetic factors on durable vaccine-induced immunity (L2003.88; L2003.102). Last but not least, this study may give us some important insights into the feasibility of adolescent vaccination trials; hopefully in the near future we will be planning such studies to evaluate HPV, HSV and even HIV vaccines in this age-group.

We attach a modified consent form and budget (significantly reduced compared to the original vaccine efficacy proposal) and look forward to your support.

Yours sincerely,

Marianne van der Sande, Hilton Whittle, Omar Sam, Mary Dowling, Pauline Kaye, David Jeffries, Maimuna Mendy, Debbi Shipton, Steve Kaye, Branwen Hennig, Giorgio Sirugo, Ebrima Bah, Abi Kahn, Adrian Hill, Pierre Hainaut, Andy Hall, Sam McConkey.

References:

-The Gambia Hepatitis Study Group. The Gambia Hepatitis Intervention Study. *Cancer Research* 1987;**47**:5782-87.

-Jack AD, Hall AD, Maine N, Mendy M, Whittle HC. What level of hepatitis B antibody is protective? *J Infect Dis* 1999;**179**:489-92

-Viviani S, Jack A, Hall AJ *et al*. Hepatitis B vaccination in infancy in The Gambia: protection against carriage at 9 years of age. *Vaccine* 1999;**17**:2946-50

-Whittle HC, Jaffar S, Wansborough M *et al.* Observational study of vaccine efficacy 14 years after trial of hepatitis B vaccination in Gambian children. *BMJ* 2002;**325**:569-73.

-Williams I *et al.* Long term antibody response to hepatitis B vaccination beginning at birth and to subsequent booster vaccination. *Pediatr Inf Dis J* 2003;**22**:157-63.

MRC/Gambia Government/WHO-IARC	SCC/EC 886/842 – L2003/64-88-xxx

Version 2	Draft 30112003

**Participant Information Sheet**

**Gambia Hepatitis Vaccine Efficacy Study**

**What is the problem with hepatitis B?** Hepatitis B is an infection of the liver. It is common in The Gambia. Most people who get infected do not get sick when this happens. However, in some people the infection persists, and becomes chronic. This may lead to liver diseases many years later. The infection can be prevented through vaccination.

**What is this study about?** Between 1986 and 1990, the Gambian Government, the Medical Research Council and the International Agency for Research on Cancer, started a trial of hepatitis B vaccine. In this period, hepatitis B vaccine was gradually included in the routine vaccinations. Vaccinations given to all children born in that period have been recorded. Over the past years, we have done several follow-up studies to look at how well the vaccine protected after 4 and 9 years of age. This study will determine if protection against infection is still good after 15 years as this would help the Government decide if an additional dose of vaccine is necessary.

**Why are we asking you to participate?** We think you may have been part of the group of people of whom we have a record of the vaccinations. We are asking you to participate in a study to see how well the vaccine has been protecting people after 15 years, and if a booster dose would be needed.

**What happens if you agree to participate?** If you agree, we will first ask you and your guardian(s) some questions. If you still have your infant welfare card, we would like to copy some details from it. We would also like to examine your arms for the presence of a BCG scar. All this will help us finding your vaccination record in our files. If we find your vaccination file, we will come back to you. If you are still willing to participate, we will collect a small amount of blood (5mls, approximately 1 teaspoon). W we will use this to study how well the vaccinations have been protecting you from infection and to study the way you have responded to the vaccine. A few drops might be used to find out if the genes (DNA) you were born with have played a role in protecting you from the infection. If we find you are carrying the virus, we will come back to you and confirm this is the case in a second sample. If you were vaccinated, we may give you a booster dose, this will be decided by chance. If you receive a booster dose, we will request for another 5 ml sample two weeks later to see how well your body responded. If you were vaccinated, regardless of whether or not your received a boost, we will come back and ask for another 5ml sample after one year. If you didn’t receive a boost at the start, you will be offered a boost then. If you were never vaccinated, and you are still at risk of infection, you can receive a full vaccination course.

If you have any medical problem during the study period, you can receive available care free of charge at the MRC.

**Questions?** Please feel free to ask any questions you want at any time during the study. You can also call Dr Marianne van der Sande on 497861. All personal data collected for this study will be treated as confidential information. You are free to leave the study at any time you so wish. This will not affect your access to normal medical care. If you are willing to help us with this study, we would like you (and your legal guardian if indicated) to sign the consent form once we have found your records in our vaccination file.

Thank you very much.

MRC/Gambia Government/WHO-IARC	SCC/EC 886/842 – L2003/64-88-xxx

Version 2	Draft 30112003

### CONSENT FORM

**Gambia Hepatitis Vaccine Efficacy Study**

I, ………………………………………………………………………………(name) , nr ………

declare that the above study has been explained to me and that I willingly and voluntarily agree to participate. I understand I have the right to withdraw from the study whenever I so desire, without affecting my right to normal medical care.

Signature or thumbprint.....................................................................Date |__|__|-|__|__|-|2|0|0|__|

*(if applicable):*

I, …………………………………………………………………………….……(name)

guardian (…………………………………(specify))

of the above, declare that the above study has been explained to me and I give permission for her/him to participate. I understand s/he has the right to withdraw from the study whenever s/he so desires, without affecting the right to normal medical care

Signature or thumbprint.....................................................................Date |__|__|-|__|__|-|2|0|0|__|

I, …………………………………………………………………………………(name),

field worker, declare that I have explained the study to the above participant and her/his guardian, that as far as I am aware they have understood well what participation entails and that they freely and willingly give their consent to participate.

Signature	.................................................................…..Date |__|__|-|__|__|-|2|0|0|__|
